# Prognostic Role of Tumor Mutational Burden in Cancer Patients Treated With Immune Checkpoint Inhibitors: A Systematic Review and Meta-Analysis

**DOI:** 10.3389/fonc.2021.706652

**Published:** 2021-07-29

**Authors:** Taobi Huang, Xia Chen, Huiyun Zhang, Yuan Liang, Longquan Li, Hui Wei, Weiming Sun, Yuping Wang

**Affiliations:** ^1^Department of Gastroenterology, The First Clinical Medical College, Lanzhou University, Lanzhou, China; ^2^Department of Gastroenterology, The First Hospital of Lanzhou University, Lanzhou, China; ^3^Key Laboratory for Gastrointestinal Diseases of Gansu Province, The First Hospital of Lanzhou University, Lanzhou, China; ^4^Department of Endocrinology, The First Hospital of Lanzhou University, Lanzhou, China

**Keywords:** tumor mutational burden, cutoff, immune checkpoint inhibitor, overall survival, progression-free survival

## Abstract

**Purpose:**

Immunotherapy is regarded as the most promising treatment for cancer. However, immune checkpoint inhibitors (ICIs) are not effective for all patients. Herein, we conducted a systematic review and meta-analysis to explore whether tumor mutational burden (TMB) can be used as a potential prognostic biomarker for cancer patients treated with ICIs.

**Methods:**

We systematically retrieved relevant literature published in the PubMed, Embase, Web of Science, and Cochrane databases up to December 28, 2020. All cohort studies and clinical trials that reported hazard ratios (HRs) for overall (OS) and progression-free survival (PFS), as well as the corresponding 95% confidence intervals (CIs) of high and low TMB patients, were included. All statistical analyses were performed using the R software.

**Results:**

Pooled results from a total of 32 studies with 6,131 participants showed significantly increased OS (HR: 0.61, 95% CI: 0.53–0.71; *P <*0.01) and PFS (HR: 0.51, 95% CI: 0.44–0.60; *P <*0.01) for the high TMB group receiving ICIs as compared to the low TMB group. Particularly, results were found to be more significant in studies with larger sample sizes (≥30), Western patients, higher TMB cutoff values (≥20 mut/Mb), anti–PD-1 therapy, and when the sample source was tissue and tumor type was either melanoma, small cell lung cancer, or gastric cancer.

**Conclusion:**

TMB is a promising independent prognostic biomarker for cancer patients receiving ICIs, which could provide a new potential therapeutic strategy for high TMB patients who have failed traditional therapy. Furthermore, consistency in the key aspects of TMB assessment is expected in the future.

**Systematic Review Registration:**

[https://www.crd.york.ac.uk/PROSPERO], Prospective Register of Systematic Reviews (PROSPERO), identifier: CRD42021229016.

## Introduction

Worldwide morbidity and mortality rates in cancer continued to rise rapidly in 2020 (1). Although the survival time of advanced cancer patients has been significantly prolonged by a combination of multiple therapies based on chemotherapy, their prognosis remains poor. However, precision medicine development and next-generation sequencing (NGS) application have provided an opportunity to search for new predictive biomarkers in cancer.

Commonly, mutation rates increase in different types of cancer patients. The total number of mutations per megabase (excluding synonymous mutations) in tumor tissue is called the tumor mutational burden (TMB), which reflects the overall burden of tumor antigens. The key point of immunotherapy is to arouse and strengthen the host’s immune system to kill the tumor. Theoretically, the higher the TMB or mutation rate in a cancer cell, the more likely it is to be recognized by the immune system, consequently improving immunotherapy efficacy. Of the treatment modalities for cancer, immunotherapy is regarded as the most promising. In fact, cancer patient prognoses have been found to be dramatically improved by the development of immune checkpoint inhibitors (ICIs), such as the blockade of programmed cell death protein 1 (PD-1), programmed death-ligand 1 (PD-L1), and cytotoxic T-lymphocyte-associated protein 4 (CTLA-4) (2, 3). It should be noted, however, that ICIs may sometimes show negative effects, suggesting that they may only work in specific cancer types. Generally, untreated patients with high TMB tend to have poorer prognoses than patients with low TMB, but the use of ICIs has reversed this situation. Many studies, in particular, have provided evidence that non-small cell lung cancer (NSCLC) and melanoma patients with higher TMB are more likely to benefit from ICIs than those with lower TMB (4–7). Despite this, there were also studies showing no connection between TMB and the survival of patients treated with ICIs, with others even reporting the opposite correlation (8–10). In the last two years, new research has emerged in breast cancer, gastric cancer, and Merkel cell carcinoma (MCC) (11–13). Similar to previous immunotherapy studies, inconsistencies were also observed among these studies, which may have resulted from different tumor types, ethnicities, sample sources, ICIs, and research designs. Recently, the variability and consistency of TMB estimates, which may have a vital impact on predicting ICI treatment efficacy, have garnered particular interest. This variability was seen in a study comparing whole exome sequencing (WES) data from 11 laboratories showed that TMB variability increased with an increase in TMB, which had greater variability in uterine, bladder, and colon cancers than in lung and head and neck cancers (14). In a study on hepatocellular carcinoma, the TMB observed was higher in Chinese patients and stored samples than in Western patients and fresh samples (15). Moreover, TMB was found to be different in different types of biliary cancers (16).

Despite all these findings, the correlation between TMB and survival benefit in patients receiving ICIs remain uncertain due to the various results of current studies, most of which target a single type of tumor instead of pan-cancer analysis. Furthermore, the TMB threshold values varied in different studies, and previous meta-analyses did not reveal the optimal TMB threshold as a biomarker for predicting ICI reactivity, with most meta-analyses only referring to NSCLC and melanoma (17–20). In this study, a systematic review and meta-analysis of existing clinical studies was performed to evaluate whether TMB could be a potential biomarker for predicting the survival of patients receiving ICIs, explore the predictive efficacy of TMB in as many tumor types as possible, and determine the optimal TMB threshold for clinical application.

## Materials and Methods

This systematic review and meta-analysis was conducted in accordance with the Preferred Reporting Items for Systematic Reviews and Meta-Analyses guidelines (**Supplementary Table 1**). The protocol was registered on the International Prospective Register of Systematic Reviews (registration number: CRD42021229016).

### Literature Research

We systematically retrieved literature published in the PubMed, Embase, Web of Science, and Cochrane databases from the date of the database’s establishment to December 28, 2020. First, the following keywords were retrieved: “tumor mutation* burden” OR “tumor mutation* load” OR “TMB.” The results were then combined with each of the following keywords: “pembrolizumab” OR “avelumab” OR “nivolumab” OR “durvalumab” OR “tremelimumab” OR “atezolizumab” OR “immunotherap*” OR “immune checkpoint inhibit*” OR “immune checkpoint block*” OR “ICI” OR “PD-1” OR “PD-L1” OR “PD-1/PD-L1” OR “anti–PD-1/anti–PD-L1” OR “CTLA-4.” The complete search strategies are listed in the **Supplementary Material** (**Supplementary Figure 1**).

### Selection Criteria

We conducted the study using the following inclusion criteria: (1) patients diagnosed with cancer using the existing gold standard; (2) tissue or blood-based TMB of patients were measured; (3) patients treated with ICIs; and (4) interesting studies that reported hazard ratios (HRs) for overall (OS) or progression-free survival (PFS), as well as the corresponding 95% confidence interval (CI) of high and low TMB patients. On the other hand, studies that met any of the following criteria were excluded: (1) patients who had not been treated with ICIs or had received treatment other than ICIs at the same time; (2) studies that did not provide sufficient information to calculate HR and 95% CI; and (3) non-human studies, review articles, conference abstracts, editorials, comments, or letters.

### Data Extraction and Quality Assessment

Data extraction was performed independently by two researchers (HTB and CX). The following information was extracted in an Excel spreadsheet: the first author, publication year, region of study, study type, tumor type, immunotherapy drug, TMB detection method, sample source, TMB cutoff value, number of patients with high/low TMB, and corresponding HRs and 95% CIs of OS and PFS.

The Newcastle-Ottawa Scale (NOS) and Methodological Index for Non-randomized Studies (MINORS) were used to assess the risk of bias in cohort studies and single-arm clinical trials, respectively (21, 22). Using the NOS assessment tool, we graded the selection of exposure, comparability of the study group, and outcome for each study, with eight subitems. Studies with ≥6, 4–5, and ≤3 stars were considered to have low, moderate, and high risk of bias, respectively. In the MINORS assessment tool, eight items, referring to aim, patients, data collection and calculation, selection and assessment of endpoints, follow-up, and loss to follow-up of studies, were used. The items were scored as 0 (not reported), 1 (reported but inadequate), or 2 (reported and adequate).

### Statistical Analyses

All statistical analyses were performed with the R version 4.0.3 program (The R Project for Statistical Computing) using the meta package. Meta-analysis was conducted to compare OS or PFS between the high and low TMB groups, and the results were estimated using pooled HR with 95% CI, wherein an HR <1 indicated increased survival for the high TMB as compared to the low TMB group. We then tested for heterogeneity between studies using the χ_2_ test and *I^2^* statistics, in which *I^2^* values of 25%, 50%, and 75% represented low, moderate, and high heterogeneity, respectively (23). Moreover, random-effects models were used to obtain pooled HRs due to moderate and high heterogeneity, and sensitivity analyses were subsequently performed to assess the stability of the pooled effects by omitting each study sequentially.

To identify the sources of heterogeneity and analyze the factors related to clinical significance, we also performed subgroup analyses based on region of study, study type, tumor type, sample source, immunotherapy drug, TMB detection method, TMB cutoff value, and sample size. However, the subgroup analyses did not adequately explain the sources of heterogeneity. In addition, to avoid data over-interpretation, only the TMB cutoff value, sample source, and sample size were included in the meta-regression.

Finally, we evaluated publication bias using funnel plot symmetry and quantifiable Egger’s test, wherein a *p*-value <0.05 was considered to have a statistically significant publication bias in the latter.

## Results

### Study Selection and Characteristics

**Figure 1:**
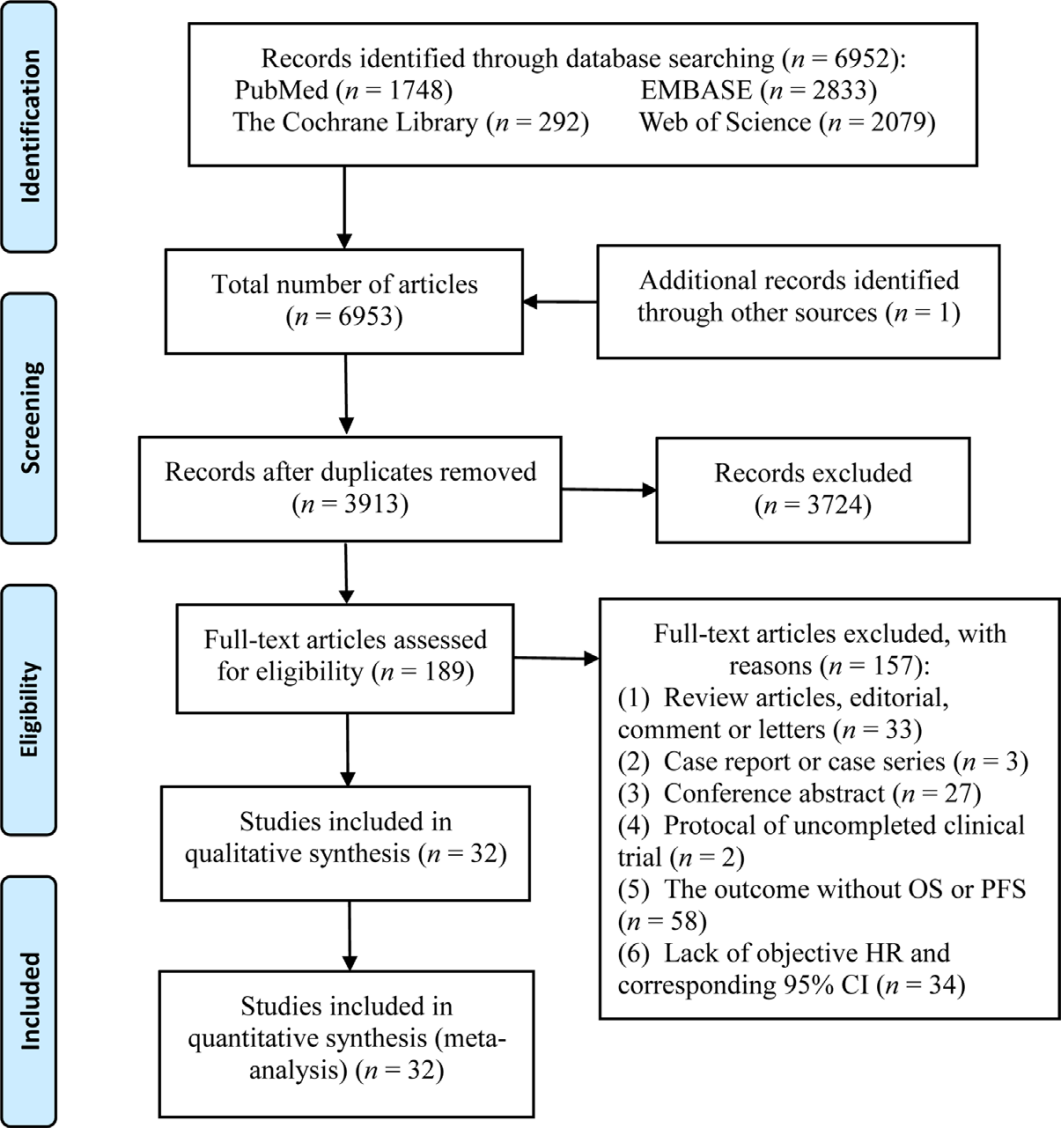
The flow diagram of the study selection process.

A total of 6,952 potential articles were identified following preliminary retrieval. After removing duplicates and reviewing the title, abstract, and the full text, 32 studies were finally included in the systematic review and meta-analysis. A flow diagram of the study selection process is presented in **Figure 1**.

A total of 32 studies with 6,131 participants were published between 2016 and 2020, with the number of patients in the individual studies ranging from 13 to 1,662. These included studies were noted to be from eight different countries in North America, Asia, and Europe. The top two countries that contributed the greatest number of articles and sample sizes were the USA and China, which included 10 clinical trials, 9 prospective cohort studies, and 13 retrospective cohort studies. The types of tumors in these studies referred to multiple solid tumors (n = 4), NSCLC (n = 16), melanoma (n = 4), gastric cancer (n = 2), small cell lung cancer (n = 3), urothelial cancer (n = 1), breast cancer (n = 1), MCC (n = 1), and head and neck squamous cell carcinoma (n = 1). The immunotherapies used in these studies were diverse ICIs, including anti–PD-1, anti–PD-L1, and anti-CTLA4 therapies. Characteristics of each study are presented in **Table 1**.

**Table 1:** The characteristics of the studies included in the meta-analysis.

Study	Year	Country	Study Type	Tumor Type	Immunotherapy Drug	Sample Source	TMB Detection Method	TMB Cutoff (mut/MB)	No. Patients		Outcome	QA
									H	L		
Li et al. (24)	2020	China	RCS	Melanoma	P	NR	NR	≥2	10	11	PFS	4
Aggarwal et al. (25)	2020	USA	Clinical trial	NSCLC	P	Blood	NGS	≥16	Total 26		OS; PFS	11
Alborelli et al. (26)	2020	Switzerland	RCS	NSCLC	ICIs	Tissue	NGS	≥9	25	51	OS; PFS	6
Joshi et al. (27)	2020	USA	PCS	Urothelial cancer	Anti–PD-1/L1	NR	FoundationOne	NR	Total 34		OS	6
Wang et al. (28)	2020	China	PCS	NSCLC	Anti–PD-1/L1	Blood	FoundationOne	≥6	28	36	OS	9
								≥16	103	326	OS
Wang, Z et al. (29)	2019	China	PCS	NSCLC	Anti–PD-1/L1	Blood	CGP	≥6	28	22	PFS	8
Chae et al. (8)	2019	USA	RCS	NSCLC	Anti–PD-1/L1	Blood	NGS	Median	Total 20		OS; PFS	7
									Total 22		OS; PFS
Gogas et al. (5)	2020	Greece	PCS	Melanoma	P	Tissue	FoundationOne	≥10	Total 224		PFS	7
Fang et al. (30)	2019	China	RCS	NSCLC	Anti–PD-1/L1	Tissue	WES	157	25	48	PFS	6
							NGS	10	26	49	PFS
Goodman et al. (31)	2019	USA	PCS	Multiple tumors	Anti–PD-1/L1 or anti-CTLA4	Tissue	FoundationOne	≥20	15	45	OS; PFS	7
B-S et al. (11)	2020	USA	Clinical trial	Breast cancer	ICIs	Tissue	OncoPanel	6	12	50	OS; PFS	12
Goodman et al. (32)	2017	USA	RCS	Multiple tumors	Multi-Immunotherapy	Tissue	FoundationOne	≥20	38	113	OS; PFS	8
Hodi et al. (33)	2019	USA	RCS	Melanoma	N	Tissue	NR	NR	23	30	OS; PFS	/
					N				95	97	OS; PFS
					I				101	93	OS; PFS
					N + I				94	103	OS; PFS
D'Angelo et al. (13)	2020	USA	Clinical trial	MCC	Avelumab	Blood and Tissue	WES	≥2	11	25	OS; PFS	9
Davis et al. (34)	2018	USA	RCS	NSCLC	ICIs	Blood	NGS	NR	Total 19		OS	/
									Total 18		PFS
Kowanetz et al. (9)	2016	USA	Clinical trial	NSCLC	A	Tissue	FM1 panel	16.6	Total 367		OS; PFS	/
Ricciuti et al. (35)	2018	USA	RCS	SCLC	Anti–PD-1 ± anti–CTLA-4	Tissue	NGS	9.29	21	23	OS; PFS	9
Griesinger et al. (10)	2017	Germany	Clinical trial	NSCLC	A	Tissue	FoundationOne	≥13.5	Total 102		OS; PFS	/
								≥17.1	Total 371		OS; PFS
He et al. (36)	2020	China	RCS	NSCLC	Anti–PD-1/L1	Tissue	TMBRB	≥10	84	243	OS; PFS	6
Yang et al. (37)	2020	USA	Clinical trial	Multiple tumors	ICIs	Tissue	NGS	≥6.88	9	94	OS	10
Shim et al. (38)	2020	Korea	PCS	NSCLC	Anti–PD-1/L1	Tissue	WES	top 25%	47	151	PFS	7
Li et al. (39)	2020	NR	Clinical trial	HNSCC	D ± T	Tissue	WES	≥upper tertile	Total 153		OS	/
					D and D+T			NR	Total 76		OS
Kim et al. (12)	2020	Korea	RCS	Gastric cancer	P/N	Tissue	NGS	≥14.31	8	55	PFS	8
Huang et al. (40)	2020	China	RCS	NSCLC	Anti–PD-1/L1	Tissue	NGS	≥10	14	20	OS; PFS	7
									7	7	OS; PFS
Wang, F et al. (41)	2019	China	PCS	Gastric cancer	Toripalimab	Blood	WES	≥12	12	42	OS; PFS	6
Ohue et al. (42)	2019	Japan	PCS	NSCLC	Anti–PD-1	Tissue	NGS	NR	Total 13		OS; PFS	6
Ricciuti et al. (43)	2019	USA	RCS	SCLC	Anti–PD-1 and/or anti–CTLA-4	Tissue	NGS	>9.68	26	26	OS; PFS	/
								>9.78	Total 52		OS
Lai et al. (44)	2019	USA	PCS	SCLC	Anti–PD-1 ± anti–CTLA-4	Tissue	NGS	upper tertile	Total 57		PFS	/
Kim et al. (45)	2018	USA	Clinical trial	NSCLC	A	Blood	NR	≥20	19	100	PFS	/
Heeke et al. (46)	2019	France	RCS	NSCLC	Anti–PD-1/L1	Tissue	FoundationOne	≥15	15	21	PFS	7
				Melanoma					15	17	PFS	
Higgs et al. (47)	2018	USA	Clinical trial	NSCLC	D+T	Tissue	FoundationOne	≥11.41	37	69	PFS	8
Samstein et al. (4)	2019	USA	Clinical trial	Multiple tumors	ICIs	Tissue	NGS	top 20%	1662		OS	9

TMB, tumor mutation burden; H, high TMB; L, low TMB; OS, overall survival; RFS, progression-free survival; HR, hazard ratio; CI, confidence interval; PCS, prospective cohort study; RCS, retrospective cohort study; P, pembrolizumab; NR, not reported; NSCLC, non-small cell lung cancer; NGS, next-generation sequencing; ICIs, immune checkpoint inhibitors; N, nivolumab; A, atezolizumab; CGP, cancer gene panel; WES, whole-exome sequencing; I, ipilimumab; MCC, Merkel cell carcinoma; SCLC, small cell lung cancer; D, durvalumab; T, tremelimumab; TMBRB,TMB radiomic biomarker; HNSCC, head and neck squamous cell carcinoma.

Of the 32 studies, the quality of 8 conference abstracts could not be evaluated, whereas the other 24 studies, including 6 clinical trials and 18 cohort studies, were of medium or high quality. The results of the quality assessment are presented in **Table 1** and **Supplementary Tables 2**, **3**.

### Comparison of OS and PFS Between High and Low TMB

**Figure 2:**
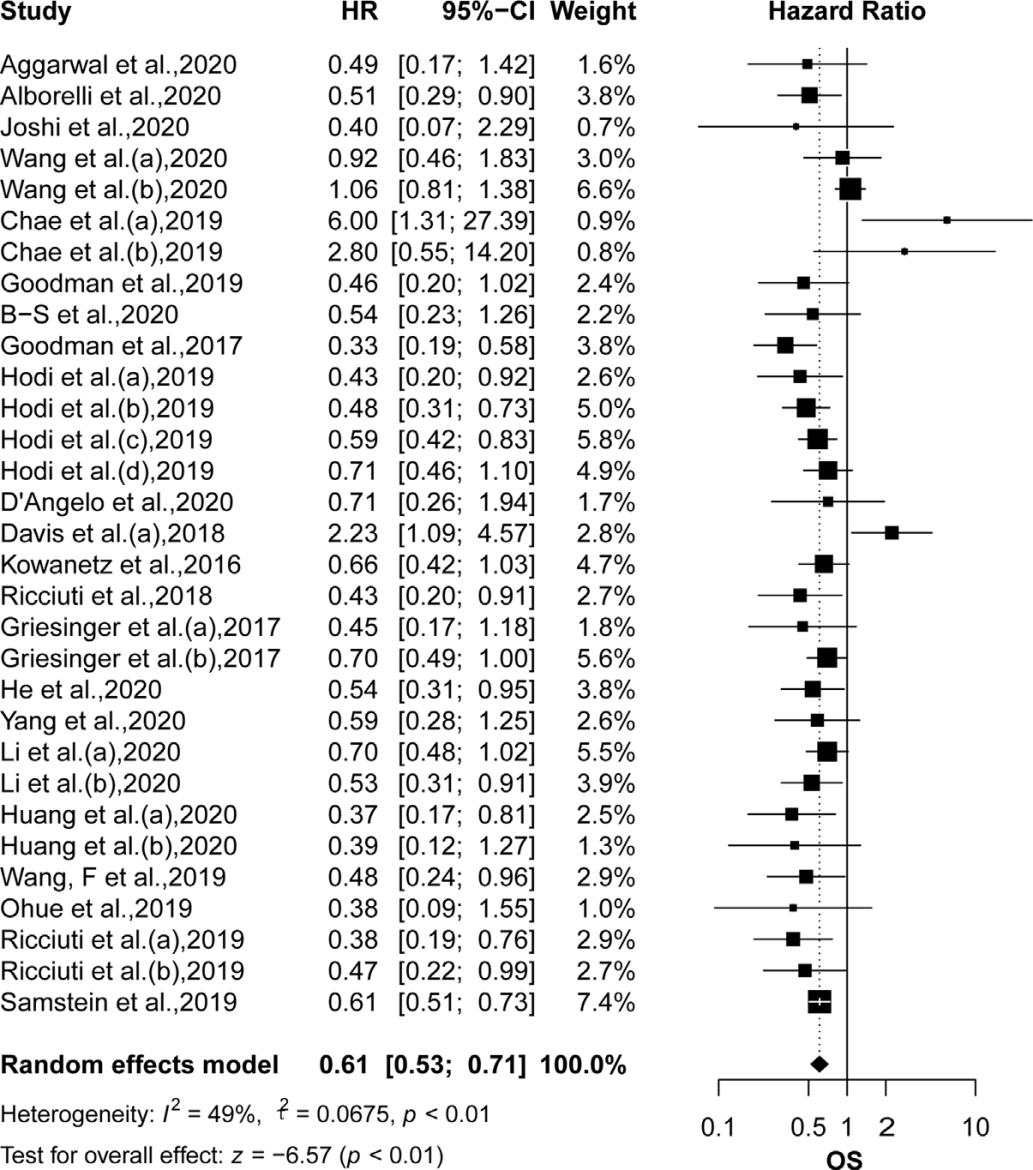
The forest plot of OS in patients with high TMB compared to those with low TMB. OS, overall survival; TMB, tumor mutational burden.

**Figure 3:**
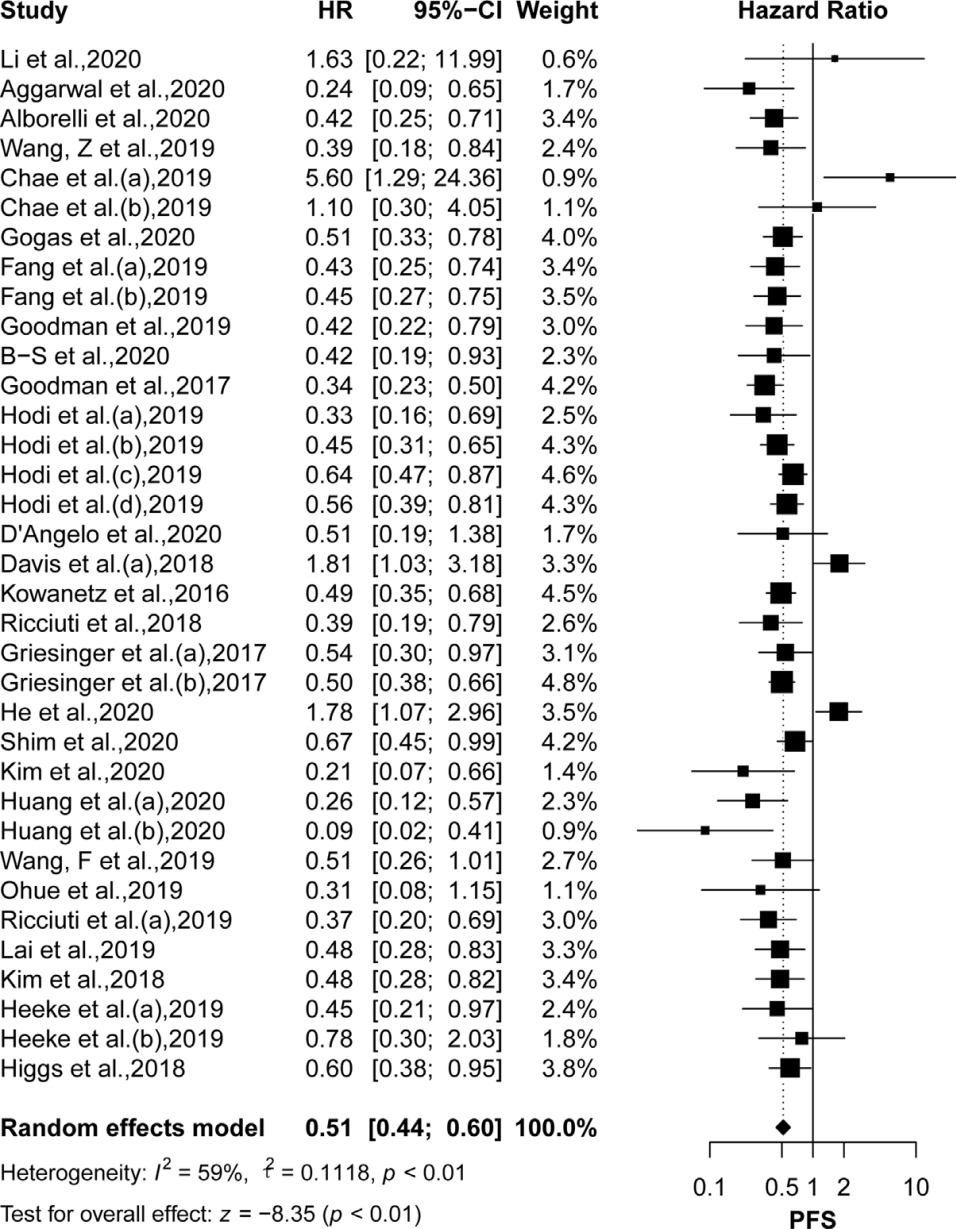
The forest plot of PFS in patients with high TMB compared to those with low TMB. PFS, progression-free survival; TMB, tumor mutational burden.

OS was reported in 22 articles, and PFS was reported in 26 articles. The pooled effects of HRs for both OS (HR: 0.61, 95% CI: 0.53–0.71; *P <*0.01) and PFS (HR: 0.51, 95% CI: 0.44–0.60; *P <*0.01) showed significantly greater benefits for the high TMB group receiving ICIs as compared to that of the low TMB group (**Figures 2** and **3**). Moreover, the results showed moderate to high heterogeneity among the combined studies (*I^2 =^*49% and 59%, respectively).

### Subgroup Analyses

**Figure 4:**
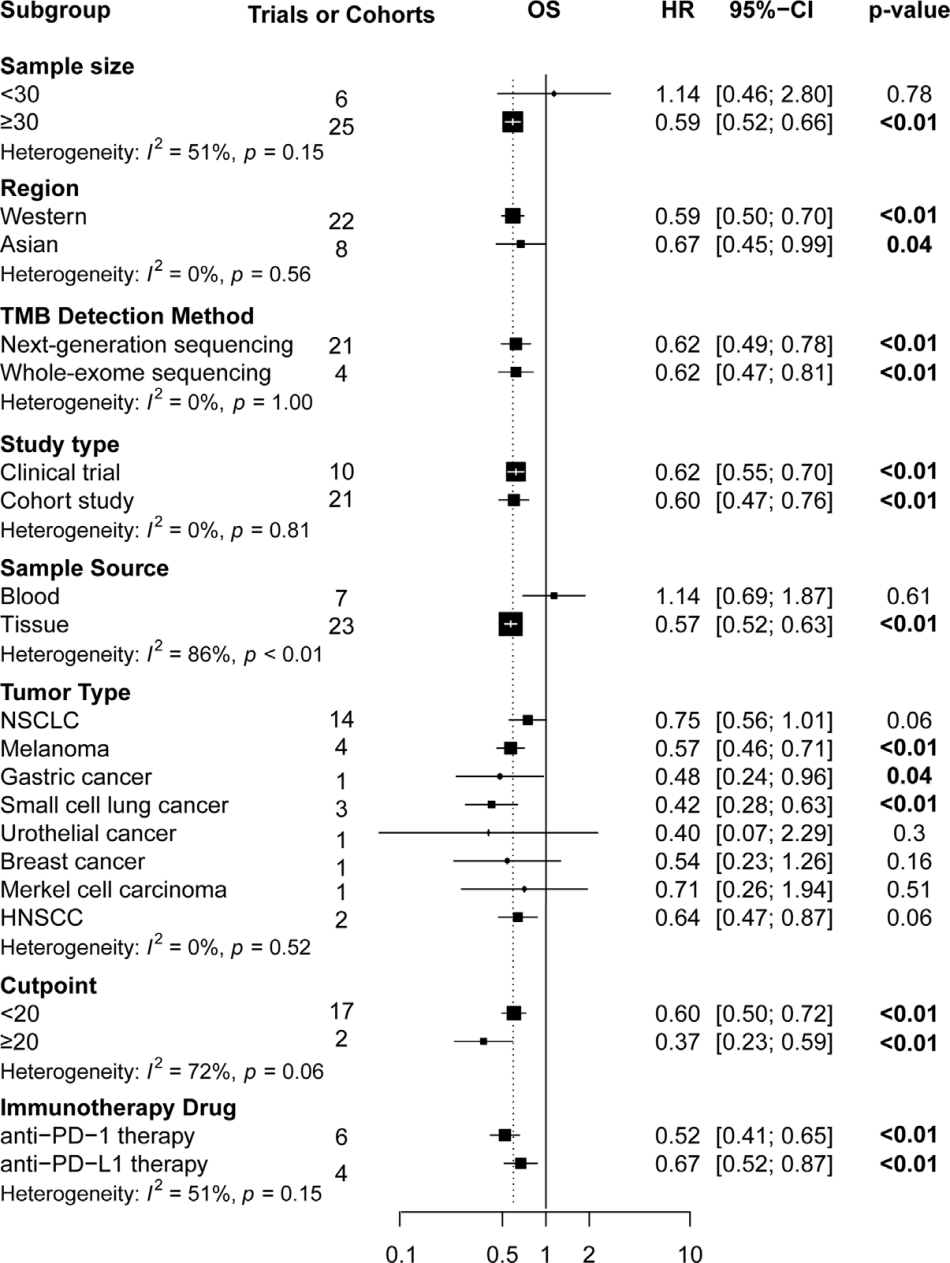
The subgroup analysis in OS of patients with high TMB compared to those with low TMB. OS, overall survival; TMB, tumor mutational burden.

**Figure 5:**
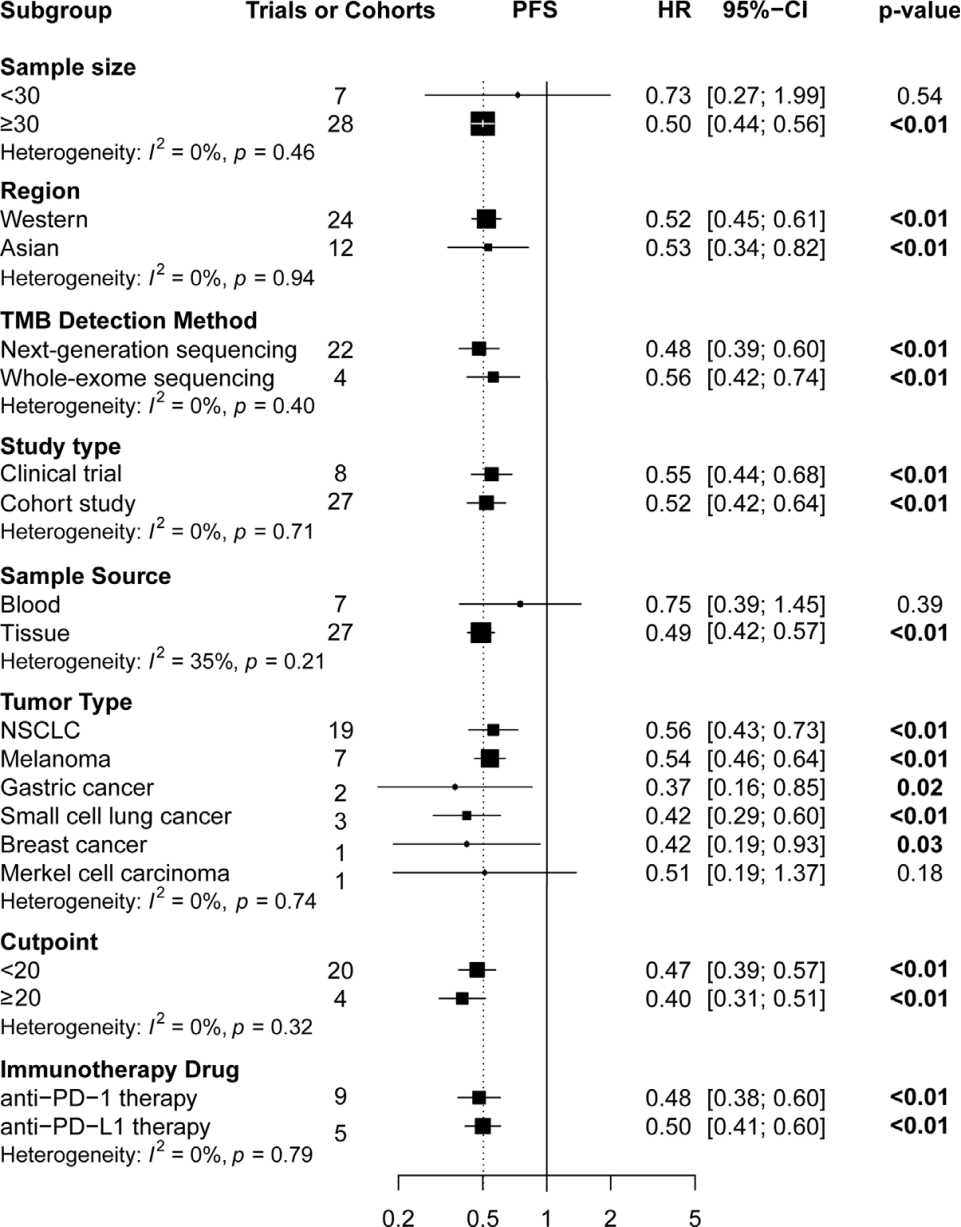
The subgroup analysis of PFS in patients with high TMB compared to those with low TMB. PFS, progression-free survival; TMB, tumor mutational burden.

Subgroup analyses (**Figures 4** and **5**) showed that Western patients (HR: 0.62, 95% CI: 0.53–0.72) with high TMB appeared to have better OS than Asian patients; however, the association of TMB level with OS and PFS had little correlation with the TMB detection method and study type. Regarding tumor type, NSCLC (HR: 0.56, 95% CI: 0.43–0.72) or breast cancer (HR: 0.42, 95% CI: 0.19–0.93) patients with high TMB had significantly better PFS, and significant correlations between high TMB, OS, and PFS benefits in gastric cancer, melanoma, and small cell lung cancer patients were found. Regarding TMB cutoff, high TMB patients seemed to have greater OS benefits when the TMB cutoff value was ≥20 mut/Mb (HR: 0.37, 95% CI: 0.23–0.58) compared with a cutoff value of <20 (HR: 0.60, 95% CI: 0.50–0.73). Regarding ICI use, high TMB patients receiving anti–PD-1 therapy seemed to have greater OS benefits (HR: 0.52, 95% CI: 0.41–0.65) than those receiving anti–PD-L1 therapy (HR: 0.67, 95% CI: 0.51–0.86). However, high TMB patients had no increased OS or PFS benefit compared to low TMB patients in studies with sample sizes of <30 and when the sample source was blood or the tumor type was MCC. Additionally, urothelial cancer patients with high and low TMB did not have significantly different OS.

### Meta-Regression Analyses

According to the meta-regression of univariate analyses, OS heterogeneity may be ascribed to sample size (p = 0.0082), sample source (p < 0.0001), and TMB cutoff value (p = 0.0963). Following multivariate analyses, the sample source and TMB cutoff value could be considered as the source of heterogeneity. As for PFS, only sample size could explain some of the sources of heterogeneity (**Table 2**).

**Table 2:** Univariate analyses and multivariate analyses of meta-regression.

Covariate	Univariate analyses				Multivariate analyses			
	Moderators	I^2^ (residual heterogeneity)	Residual Heterogeneity	Signif.	Moderators	I^2^ (residual heterogeneity)	Residual Heterogeneity	Signif.
OS								
sample size	p = 0.0082	42.51%	p = 0.0081	**	p = 0.1990			
Sample Source	p < 0.0001	12.34%	p = 0.2769	***	p = 0.0004	0.00%	0.7883	***
Cutoff of TMB	p = 0.0963	29.80%	p = 0.21137	.	p = 0.0911			.
PFS								
sample size	p = 0.0495	55.67%	p < 0.0001	*	p = 0.0273			*
Sample Source	p = 0.0756	58.72%	p < 0.0001	.	p = 0.7556	44.82%	0.0163	
Cutoff of TMB	p = 0.4601	47.51%	p = 0.0064		p = 0.3136			

Signif. codes: ‘***’, 0.001; ‘**’, 0.01; ‘*’, 0.05; ‘.’, 0.1; ‘ ’, 1.

### Sensitivity Analysis and Publication Bias

Sensitivity analyses revealed that the pooled effects scarcely changed, regardless of which study was excluded (**Supplementary Figures 2** and **3**). Funnel plots were symmetric (**Supplementary Figure 4**), and the *p*-value of Egger’s test was 0.4789 for OS and 0.7120 for PFS, suggesting no significant publication biases.

## Discussion

This systematic review and meta-analysis including 32 studies with 6,131 participants strongly suggested that both OS and PFS of the high TMB group receiving ICIs were significantly better than those of the low TMB group, which was in agreement with a previous study (17). Furthermore, this is the first study to analyze the prognostic value of TMB in cancer patients receiving ICIs in terms of cutoff values. This benefit to high TMB patients depended on the existence of ICIs, since no survival benefit was observed in these patients who did not receive ICI treatment (4). Although the combined effects had moderate to high heterogeneity, the survival benefit of high TMB patients was consolidated using sensitivity and meta-regression analyses. Sensitivity analysis results indicated robustness, and the sample source and TMB cutoff value were proven to be possible sources of heterogeneity for OS, after meta-regression analyses, whereas for PFS, the meta-analysis did not fully explain the source of heterogeneity.

In subgroup analyses, survival benefits were found to be more significant in studies with large sample sizes; in Western patients; in those using anti–PD-1 therapy; when the sample source was tumor tissue; when the tumor type was either melanoma, small cell lung cancer, or gastric cancer; and when the TMB cutoff value was ≥20 (mut/MB). In contrast, when the sample size <30, the sample source was blood, and the tumor type was either MCC or urothelial cancer, no significantly increasing benefits in high TMB patients were found.

In our review, studies with a sample size of <30 comprised <10% of all included studies, with relatively poor credibility; thus, they can be ignored. However, the overall outcome did not change. After subgroup analysis, only the heterogeneity of studies with a larger sample size (≥30) decreased significantly, and Western patients with high TMB seemingly had a better OS than Asian patients. Aside from race, this result may also be related to the small sample size of Asian studies, different types of ICIs, and differences in TMB detection methods and research designs.

In previous studies, TMB was measured using WES. With the development of precision medicine, next-generation targeted gene panel sequencing has been gradually applied in TMB detection due to its more timely and economic advantages (48). Our results showed that there was no statistical difference between the two TMB values measured using WES and NGS in predicting the prognosis of patients receiving ICIs. Inclusive studies using the blood-based TMB detection method also did not show better benefits from ICIs in high TMB patients, which may be related to the disadvantages of the technique. For example, blood samples do not have sufficient circulating DNA, and they lack specific types of mutations in tumor tissues. However, blood-based TMB detection is a feasible option for patients who are unable to undergo biopsy or obtain tissue samples from them (49, 50). Therefore, whether blood-based TMB detection can effectively identify patients who can benefit from immunotherapy requires further prospective studies.

To date, neither the TMB calculation method nor the threshold for reaching “high” TMB has been consistent (51). When calculating TMB, some studies calculate the total number of non-synonymous mutations in each coding region, while others only count mutations that can cause protein changes. Consequently, TMB thresholds related to survival benefits varied greatly in the different studies included in our meta-analysis. A clinical trial referring to multiple tumor types, for one, showed that a higher cutoff value was attributed to a more significant increased OS in high TMB patients (4). In the subgroup analyses of our review, the combined effects of the cutoff value of ≥20 mut/MB were more significant than those of <20 mut/MB. Despite this, simply defining a certain threshold value as “high TMB” was not suitable for predicting the effect of immunotherapy for each type of tumor, as confirmed in a new study (52). In our study, the heterogeneity of different studies on the same tumor type, such as having different TMB detection methods and participants from different populations, limited our search for the best cutoff value for each tumor type. For the different types of cancer, determining the “high” TMB threshold requires more clinical research and statistics based on a large amount of patient information. Even so, it remains to be seen whether it is applicable to clinical practice, since TMB detection has a guiding significance in immunotherapy strategies. In the future, to better realize TMB as a powerful predictive biomarker, it is necessary to determine the best TMB cutoff values for different tumor types.

Moreover, previous studies revealed that the survival benefits of high TMB patients did not depend on PD-L1 expression, wherein patients with high TMB and PD-L1 positivity were found to be nearly two separate populations, even when TMB was better than PD-L1 expression as a biomarker for predicting ICI efficacy (41, 53). The combined detection of many kinds of biomarkers, such as TMB, copy number alteration, and T-cell activity indexes, will be more necessary and effective for screening patients who are most likely to benefit from them (7, 54).

Furthermore, anti–PD-1/PD-L1 and anti–CTLA-4 combination therapies or the addition of other treatments also showed surprising results (47). In fact, the Food and Drug Administration has approved the combination of nivolumab and chemotherapy as a first-line immunotherapy for advanced gastric cancer (55). Aside from this, new types of immuno- therapies are also being used and developed. A clinical trial, in particular, showed that the objective remission rate of multiple myeloma patients can reach 100% after chimeric antigen receptor-T cell therapy (NCT03548207). Therapeutic cancer vaccines have also significantly prolonged disease-free survival in breast cancer patients (56). However, even with these findings, more prospective studies are needed to verify whether our results are applicable to the aforementioned patients.

Despite the results of our review, certain limitations of this study should be considered. First, the HRs and corresponding 95% CIs were not reported in some studies, and we could not obtain their original data; thus, these studies were excluded. This may have led to a potential publication bias. Second, since few studies have reported the incidence of adverse effects, this study no longer explored these incidences. Third, the literature included in the study was limited to English publications, which may have omitted studies in other languages that may have significant data for our review as well. Fourth, the TMB cutoff value varied from study to study, which may have led to imprecise pooled effects. Fifth, not all studies reported all their subgroup factors, therefore only the effects of those studies that reported a certain number of subgroup factors were combined in the subgroup analysis, which may have caused inaccurate identification of factors contributing to heterogeneity. Lastly, regarding PFS, the source of heterogeneity was not fully explained, which may be affected by unreported confounding factors in these studies, such as differences in sample processing, sequencing panel size, and gene coverage (14).

Overall, the results of this study fully revealed the urgent need for consistency in TMB evaluation based on panel sequencing in the future. For example, performing a sample selection from fresh tumor tissues, having a consistent TMB calculation method, and following a threshold of “high” and “low” TMB for certain tumor types or subtypes of the same tumor, are all needed. In particular, our study was the first to show that high TMB patients receiving ICIs had maximum survival benefit when the TMB cutoff point was ≥20 (mut/MB). To increase the reliability of this conclusion, it is necessary to coordinate the consistency of TMB assessment in the future. Moreover, current research in this field has been mainly related to lung cancer and melanoma, whereas our study included tumor types that were not found in previous meta-analyses, such as breast cancer, gastric cancer, and MCC, although studies related to these tumor types have only been reported individually. Thus, in the future, there is an urgent need for prospective studies with larger sample sizes aimed at different tumor types, different subtypes of the same tumor, and different ethnicities. Furthermore, studies reporting the incidence of adverse effects should also be expected, whether it be the comparison of immunotherapy safety to that of traditional chemotherapy, or the tolerance of high TMB patients to that of low TMB patients. Additionally, we believe that the combined predictive efficacy of multiple biomarkers, such as TMB, copy number alteration, and T-cell activity indices, may be beneficial. Finally, TMB may be used as a predictor not only in ICI therapy, but also in new immunotherapies, such as chimeric antigen receptor-T cell therapy and therapeutic vaccines.

## Conclusions

This systematic review and meta-analysis found that patients with high TMB who received ICIs had significantly better OS and PFS than those with low TMB, especially in studies with larger sample sizes (≥30); in those including Western patients; in studies with higher TMB cutoff values (≥20 mut/Mb); in those with anti–PD-1 therapy; when the sample source was tissue; and when the tumor type was either melanoma, NSCLC, or gastric cancer. TMB is a promising independent prognostic biomarker for cancer patients receiving ICIs, as it could provide a new potential therapeutic strategy for those with high TMB and failed chemotherapy and targeted therapy. Furthermore, consistency in the key aspects of TMB assessment is expected in the future.

## Data Availability Statement

The original contributions presented in the study are included in the article/**Supplementary Material**. Further inquiries can be directed to the corresponding authors.

## Author Contributions

Conceptualization: TH. Methodology (data collection): TH and XC. Statistical analysis: TH. Writing (original draft preparation): TH. Review and editing: TH, HZ and YW. Study supervision: YL, LL, HW and WS. All authors contributed to the article and approved the submitted version.

## Funding

This work was supported by the National Key R&D Program of China (2016YFC1302201), Natural Science Foundation of Gansu Province (18JR3RA366 and 21JR1RA116).

## Conflict of Interest

The authors declare that the research was conducted in the absence of any commercial or financial relationships that could be construed as a potential conflict of interest.

## Publisher’s Note

All claims expressed in this article are solely those of the authors and do not necessarily represent those of their affiliated organizations, or those of the publisher, the editors and the reviewers. Any product that may be evaluated in this article, or claim that may be made by its manufacturer, is not guaranteed or endorsed by the publisher.

## Supplementary Material

Supplementary Table 1 The statement of PRISMA.

Supplementary Table 2 The NOS tool for assessing the risk of bias of cohort studies.

Supplementary Table 3 The MINORS tool for assessing the risk of bias of single-arm clinical trials.

Supplementary Figure 1 Search strategies.

Supplementary Figure 2 Sensitivity analysis of pooled effects for OS in the meta-analysis.

Supplementary Figure 3 Sensitivity analysis of pooled effects for PFS for the meta-analysis.

Supplementary Figure 4 The funnel plots for OS **(A)** and PFS **(B)**.
