# Supplementary material for: Prognostic Role of Tumor Mutational Burden in Cancer Patients Treated With Immune Checkpoint Inhibitors: A Systematic Review and Meta-Analysis
Source: Front Oncol. 2021 Jul 29;11:706652. doi: 10.3389/fonc.2021.706652 (PMC8358612; doi:10.3389/fonc.2021.706652)
Supplement: Supplementary Table 2 — The NOS tool for assessing the risk of bias of cohort studies. [file Table_2.docx]

**Supplementary Table 2.** The NOS tool for assessing the risk of bias of cohort studies.

| Study | Selection | | | | Comparability | Outcome | | | NOS score |
| --- | --- | --- | --- | --- | --- | --- | --- | --- | --- |
|  | Representativeness of the exposed cohort | Selection of the non-exposed cohort | Ascertainment of exposure | Demonstration that outcome of interest was not present at start of study | Comparability of cohorts on the basis of the design or analysis | Assessment of outcome | Was follow-up long enough for outcomes to occur | Adequacy of follow up of cohorts |  |
| Gogas et al.2020 | * | * | * | * | — | * | * | * | 7 |
| Wang et al.2020 | * | * | * | * | ** | * | * | * | 9 |
| Li et al.2020 | — | * | — | * | — | * | * | — | 4 |
| Joshi et al.2020 | * | — | — | * | * | * | * | * | 6 |
| Alborelli et al.2020 | — | * | * | * | — | * | * | * | 6 |
| Shim et al.2020 | — | * | * | * | * | * | * | * | 7 |
| Kim et al.2020 | — | * | * | * | ** | * | * | * | 8 |
| Huang et al.2020 | * | * | * | * | — | * | * | * | 7 |
| He et al.2020 | — | * | * | * | — | * | * | * | 6 |
| Wang, F et al.2019 | — | * | * | * | — | * | * | * | 6 |
| Ricciuti et al.2019 | * | * | * | * | ** | * | * | * | 9 |
| Ohue et al.2019 | — | * | * | * | — | * | * | * | 6 |
| Heeke et al.2019 | * | * | * | * | — | * | * | * | 7 |
| Wang, Z et al.2019 | — | * | * | * | ** | * | * | * | 8 |
| Goodman et al.2019 | * | * | * | * | — | * | * | * | 7 |
| Fang et al.2019 | — | * | * | * | — | * | * | * | 6 |
| Chae et al.2019 | * | * | * | * | — | * | * | * | 7 |
| Goodman et al.2017 | — | * | * | * | ** | * | * | * | 8 |
