# Supplementary material for: Prognostic Role of Tumor Mutational Burden in Cancer Patients Treated With Immune Checkpoint Inhibitors: A Systematic Review and Meta-Analysis
Source: Front Oncol. 2021 Jul 29;11:706652. doi: 10.3389/fonc.2021.706652 (PMC8358612; doi:10.3389/fonc.2021.706652)
Supplement: Supplementary Table 3 — The MINORS tool for assessing the risk of bias of single-arm clinical trials. [file Table_3.docx]

**Supplementary Table 3.** The MINORS tool for assessing the risk of bias of single-arm clinical trials

| Item Study | Aggarwal et al.2020 | B-S et al.2020 | D'Angelo et al.2020 | Yang et al.2020 | Higgs et al.2018 | Samstein et al.2019 |
| --- | --- | --- | --- | --- | --- | --- |
| A clearly stated aim | 2 | 2 | 2 | 2 | 2 | 2 |
| Inclusion of consecutive patients | 2 | 2 | 0 | 1 | 0 | 1 |
| Prospective collection of data | 2 | 2 | 2 | 2 | 2 | 2 |
| Endpoints appropriate to the aim of the study | 2 | 2 | 2 | 2 | 2 | 2 |
| Unbiased assessment of the study endpoint | 0 | 0 | 0 | 0 | 0 | 0 |
| Follow-up period appropriate to the aim of the study | 2 | 2 | 2 | 2 | 2 | 2 |
| Loss to follow up less than 5% | 1 | 2 | 1 | 1 | 0 | 0 |
| Score | 11 | 12 | 9 | 10 | 8 | 9 |
