# Supplementary figures and images for: Prognostic Role of Tumor Mutational Burden in Cancer Patients Treated With Immune Checkpoint Inhibitors: A Systematic Review and Meta-Analysis
Source: Front Oncol. 2021 Jul 29;11:706652. doi: 10.3389/fonc.2021.706652 (PMC8358612; doi:10.3389/fonc.2021.706652)

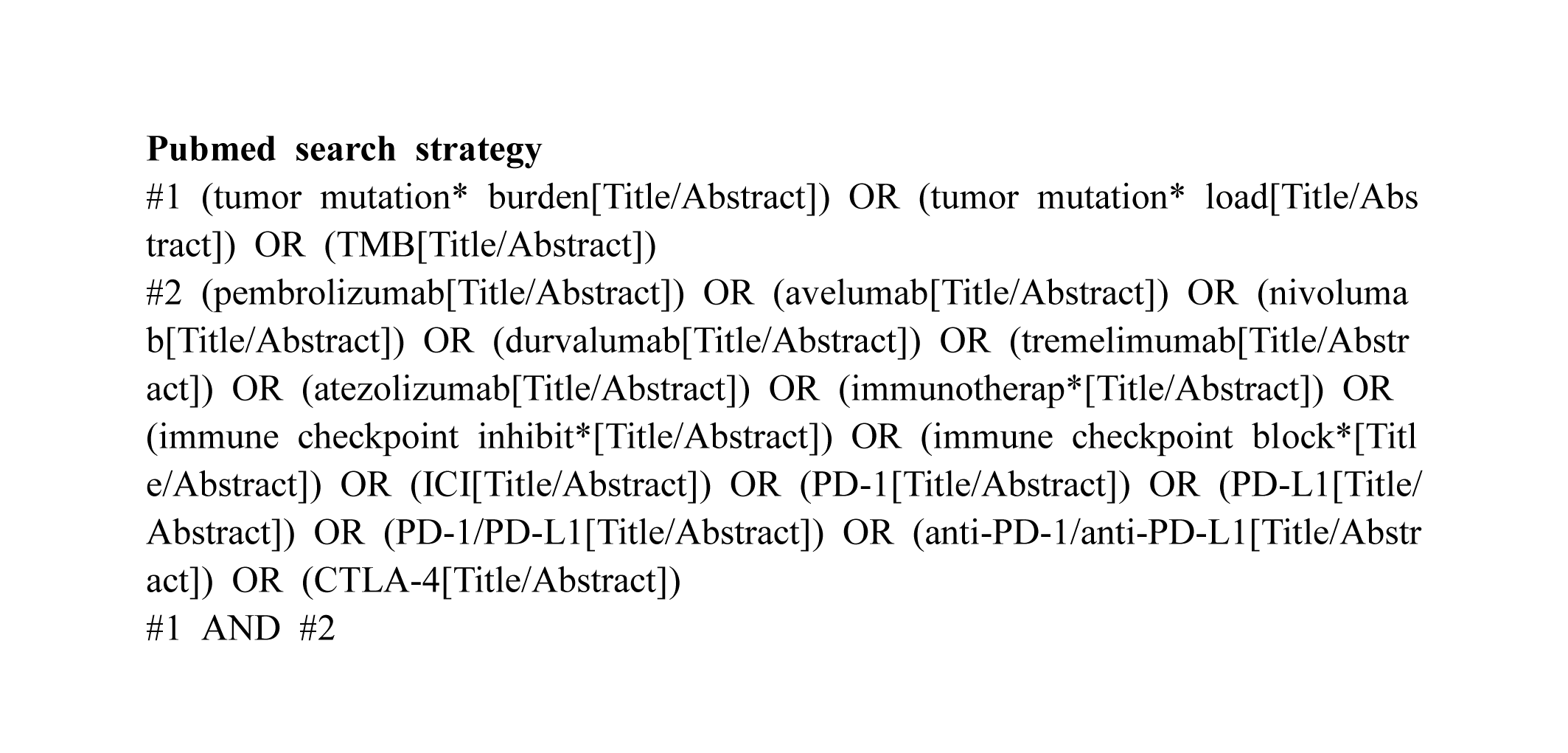

Supplement: Supplementary Figure 1 — Search strategies. [file Image_1.tif]

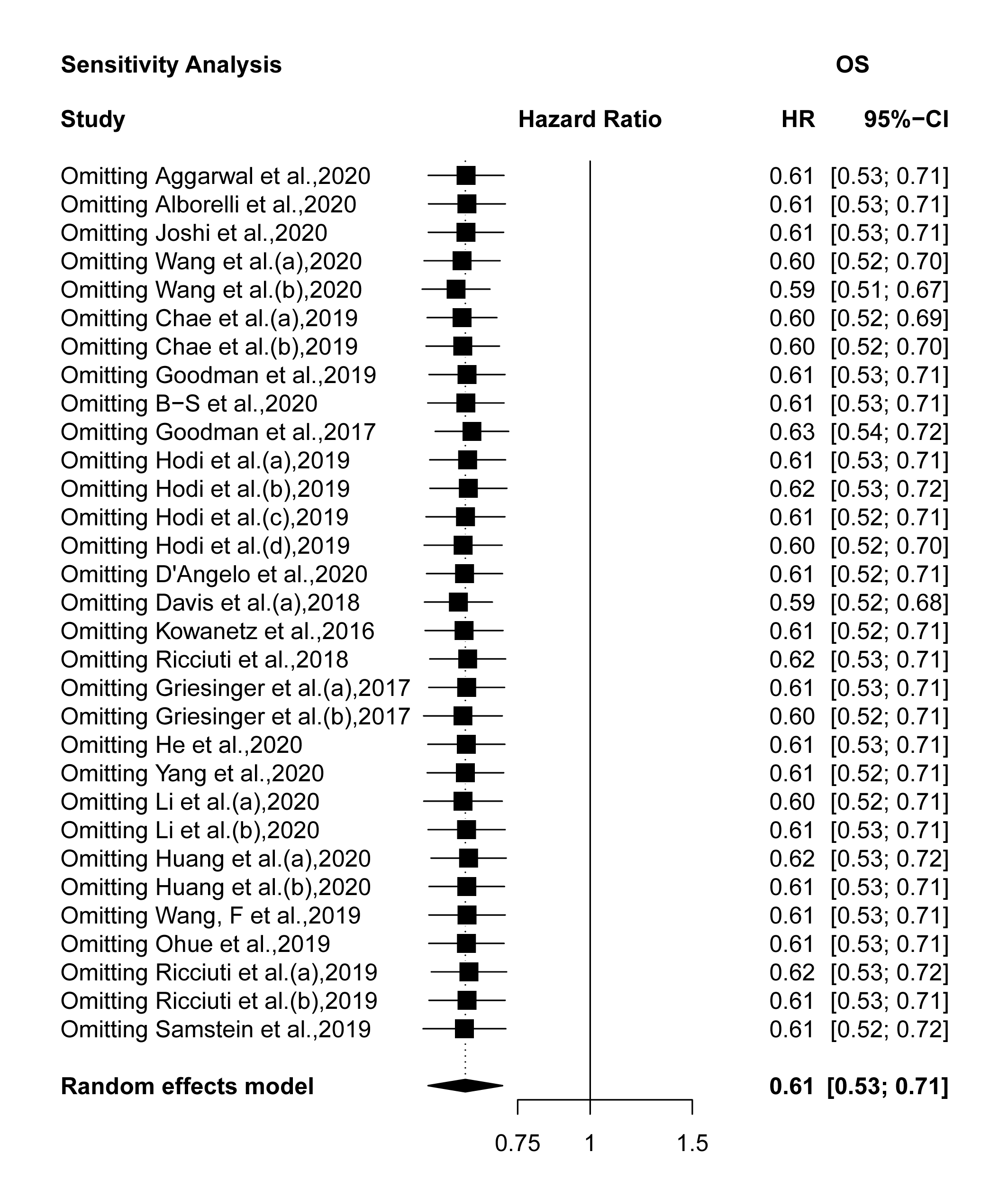

Supplement: Supplementary Figure 2 — Sensitivity analysis of pooled effects for OS in the meta-analysis. [file Image_2.tif]

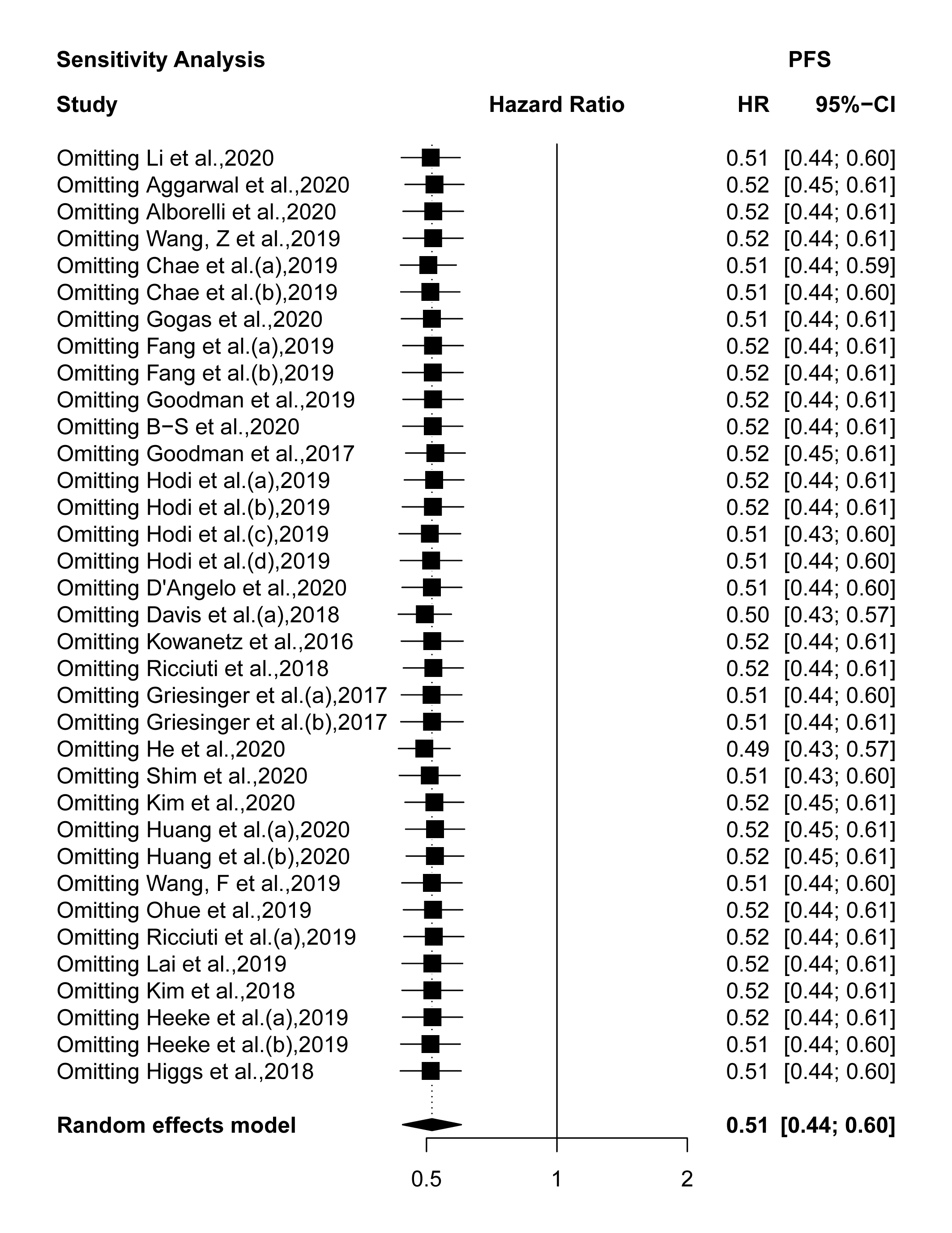

Supplement: Supplementary Figure 3 — Sensitivity analysis of pooled effects for PFS for the meta-analysis. [file Image_3.tif]

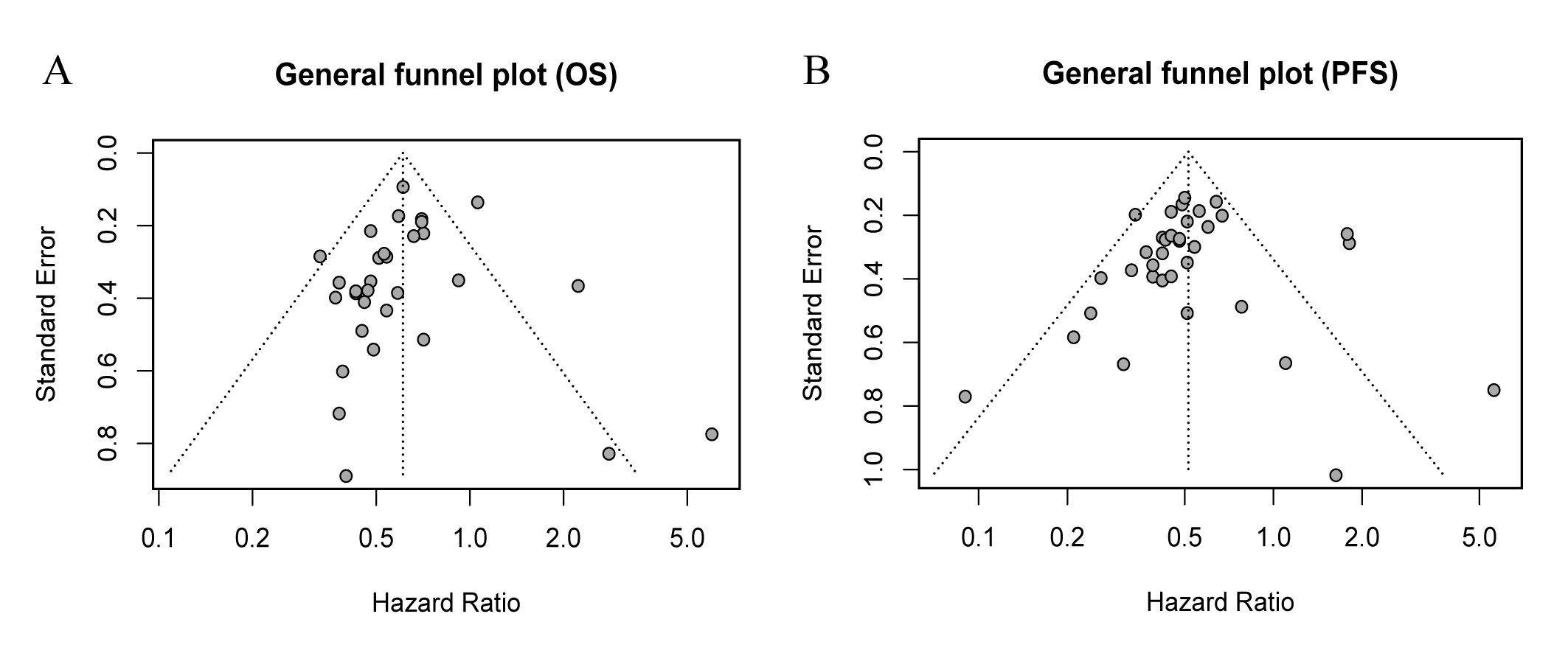

Supplement: Supplementary Figure 4 — The funnel plots for OS (A) and PFS (B). [file Image_4.tif]
